# The mitochondrial genomes of the Geometroidea (Lepidoptera) and their phylogenetic implications

**DOI:** 10.1002/ece3.9813

**Published:** 2023-02-09

**Authors:** Weili Ding, Haizhen Xu, Zhipeng Wu, Lizong Hu, Li Huang, Mingsheng Yang, Lili Li

**Affiliations:** ^1^ Finance Office Zhoukou Normal University Zhoukou China; ^2^ College of Life Science and Agronomy Zhoukou Normal University Zhoukou China; ^3^ Key Laboratory of Plant Genetics and Molecular Breeding Zhoukou Normal University Zhoukou China

**Keywords:** gene rearrangement, Geometridae, mitochondrial genome, phylogeny

## Abstract

The Geometroidea is a large superfamily of Lepidoptera in species composition and contains numerous economically important pest species that cause great loss in crop and forest production. However, understanding of mitogenomes remains limited due to relatively fewer mitogenomes previously reported for this megadiverse group. Here, we sequenced and annotated nine mitogenomes for Geometridae and further analyzed the mitogenomic evolution and phylogeny of the whole superfamily. All nine mitogenomes contained 37 mitochondrial genes typical in insects, and gene organization was conserved except for *Somatina indicataria*. In *S. indicataria*, the positions of two tRNAs were rearranged. The *trnR* was located before *trnA* instead of after *trnA* typical in Lepidoptera, whereas the *trnE* was detected rarely on the minority strand (N‐strand). This *trnR*‐*trnA*‐*trnN*‐*trnS1*‐ *trnE* ‐ *trnF* newly recognized in *S. indicataria* represents the first gene rearrangement reported for Geometroidea and is also unique in Lepidoptera. Besides, nucleotide composition analyses showed little heterogeneity among the four geometrid subfamilies involved herein, and overall, *nad6* and *atp8* have higher nucleotide diversity and Ka/Ks rate in Geometridae. In addition, the taxonomic assignments of the nine species, historically defined by morphological studies, were confirmed by various phylogenetic analyses based on the hitherto most extensive mitogenomic sampling in Geometroidea.

## Graphical abstract

In this study, we determined nine mitochondrial genomes for the Geometridae for the first time. A tRNA gene rearrangement was recognized in *S. indicataria*, which represents the first gene rearrangement reported for Geometroidea and is also unique in Lepidoptera. The mitochondrial genomes of the Geometridae were comparatively analyzed, and various phylogenetic analyses were conducted based on the hitherto most extensive mitogenomic sampling in Geometroidea, which could facilitate the understanding of Geometroidea phylogeny.

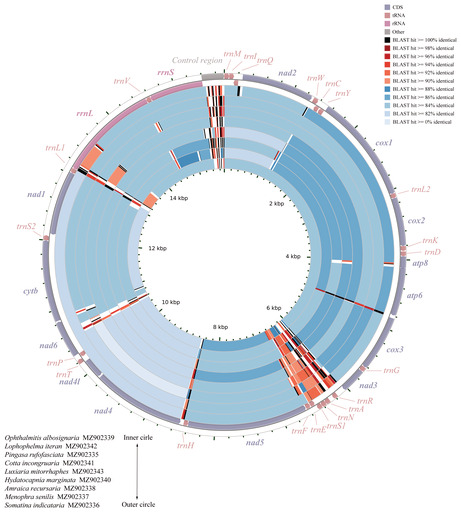

## 1 INTRODUCTION

A typical arthropod mitochondrial genome (mitogenome) is circular and generally consists of 13 protein‐coding genes (PCGs), two ribosomal RNA genes (rRNAs), and 22 transfer RNA genes (tRNAs) (Cameron, 2014; Curole & Kocher, 1999). Besides, several noncoding elements, including the control region that regulates the replication and transcription of the mitogenome, are present (Boore, 1999). The mitogenome is characterized by a series of features such as cellular abundance, absence of introns, and a lack of extensive recombination, and thus, they represent one of the important molecular markers, such as the standard *cox1* barcode sequence, used in studies on species identification and population genetics of insects, especially for the megadiverse Lepidoptera (Hajibabaei et al., 2006; Hebert et al., 2003, 2004). In recent years, with the decline of sequencing cost, increasing numbers of the whole mitogenomes have been sequenced and widely used in not only species identification and delimitation but also phylogeny and population genetics of the Lepidoptera and other insect groups (e.g., Du et al., 2019; Timmermans et al., 2014; Yang et al., 2015; Yang, Song, et al., 2019). In addition, mitochondrial gene arrangement also represents one kind of important information to infer evolutionary relationships of insects. For instance, the gene arrangement of *trnM*‐*trnI*‐ *trnQ* (the gene underlined is located on the minority strand) is regarded as a synapomorphy for Ditrysia in contrast to some other groups of Lepidoptera such as Adeloidea and Nepticuloidea and ancestral insect orders with the *trnM*‐ *trnQ* ‐*trnI* instead (Timmermans et al., 2014). However, although Lepidoptera is one of the species‐rich orders in insects, gene rearrangement events have been less reported for this group in comparison with some other orders especially the Hemiptera (e.g., Thao et al., 2004) and Hymenoptera (e.g., Tang et al., 2019) albeit with relatively lower species diversity.

The Geometroidea is one of the largest superfamilies in Lepidoptera and includes more than 24,000 described extant species (van Nieukerken et al., 2011). As leaf feeders, they feed on multiple kinds of typically woody plants, thus often causing a huge loss in agricultural and forest production (Li et al., 2018; Mitter et al., 2017). Three families, i.e., Geometridae, Uraniidae, and Sematuridae, had been defined for Geometroidea before (Mitter et al., 2017). Later, molecular evidence strongly suggested the inclusion of Epicopeiidae historically from Drepanoidea (Bazinet et al., 2013; Regier et al., 2013; Yang, Zhang, et al., 2019), and a new family Pseudobistonidae (Rajaei et al., 2015; Wang et al., 2019). To date, the relationship among the five families has been recovered as ((Uraniidae, Geometridae), (Sematuridae, (Epicopeiidae, Pseudobistonidae))) by most of the previous studies, but this topology needs to be confirmed because the clade consisting of Sematuridae, Epicopeiidae, and Pseudobistonidae has been either lowly supported (Rajaei et al., 2015; Wang et al., 2019) or sparsely sampled (Murillo‐Ramos et al., 2019) in previous studies.

In Geometroidea, mitogenomes of approximately 27 species from three families have been sequenced to date (GenBank, November 2021). This number is obviously disproportional relative to the huge species diversity of this superfamily. Moreover, among the three families, the reported mitogenomes of both the Epicopeiidae and Uraniidae were represented by one species. Given their wide application in the molecular systematics of insects, this situation will hinder the progress of investigating Geometroidea systematics using mitogenome data. Based on the existing mitogenomes, comparative analysis among geometroid members or/and deep phylogenetic analyses (e.g., Du et al., 2019; Yang et al., 2013; Yang, Song, et al., 2019) was conducted, which greatly further our understanding of phylogeny of this superfamily and related groups. In addition, the mitogenome sequences were also used to infer the phylogenetic relationships of three closely‐related *Biston* species in Geometridae, suggesting the existence of the budding speciation in these species (Cheng et al., 2017). In terms of gene arrangement, all the mitochondrial genomes published available show identical gene organization that is typical in Lepidoptera, and no gene rearrangement events have been reported for Geometroidea to date.

In this study, the mitogenomes of nine additional geometrid species were sequenced, annotated, and comparatively analyzed, aiming to increase the reported mitogenome diversity of Geometroidea and to improve our understanding of mitogenome evolution in this superfamily. Also, these data can provide mitogenome data for other studies on molecular systematics of Geometroidea. Among the nine species, *Somatina indicataria* showed a gene rearrangement of *trnR*‐*trnA*‐*trnN*‐*trnS1*‐ *trnE* ‐ *trnF* relative to the *trnA*‐*trnR*‐*trnN*‐*trnS1*‐*trnE*‐ *trnF* typical in Lepidoptera, which represents the first gene rearrangement reported for Geometroidea and is also unique in Lepidoptera.

## 2 MATERIALS AND METHODS

### 2.1 Samples, DNA extraction, and mitogenome sequencing

Adult moths were collected by light trap, at Mountain Jigongshan and Lushan country of Henan Province in China, from July to August 2020. Each specimen was identified through morphology and by blasting the standard mitochondrial *cox1* barcode on the GenBank database. After identification, nine species from Geometridae of Geometroidea were selected, of which six from Ennominae, two from Geometrinae, and one from Sterrhinae, mainly because of the high species diversity of Ennominae and lack of existing mitogenomes of Geometrinae and Sterrhinae. Detailed specimen information is shown in Table S1, and voucher specimens are deposited in the Biology Laboratory of Zhoukou Normal University, China. In phylogenetic analyses, two other mitogenome sequences were retrieved from transcriptomes of *Mania lunus* (SRR1695439) and *Calledapteryx dryopterata* (SRR1021601) available on GenBank, which represented the Sematuridae and Uraniidae, respectively, together with other available mitogenomes to perform phylogenetic analyses of the Geometroidea.

Total genomic DNA was extracted from thoracic tissue isolated from a single specimen using DNeasy tissue kit (Qiagen, Germany), following the manufacturer's instructions. Nine libraries (each for one species) were constructed with TruSeq DNA PCR‐Free Sample Preparation Kit (Illumina, United States), and sequencing was conducted using an Illumina HiSeq 2500 platform with a strategy of 150 paired‐ends.

### 2.2 Mitogenome assembly and annotation

Raw sequences were checked for quality control using the FastQC (http://www.bioinformatics.babraham.ac.uk/projects/fastqc). Then, the Adapter Removal 2 (Schubert et al., 2016) and SOAPec 2.0.1 in SOAPdenovo 2.01 software package (Luo et al., 2012) were employed to filter these raw sequences to obtain clean paired reads. Next, we assembled mitogenome from clean paired reads using the Geneious R11 (Kearse et al., 2012). In this analysis, the “map to reference” strategy was selected to map all cleaned reads to an “anchor” of standard mitochondrial *cox1* barcoding sequence that was amplified earlier using insect primer pair Lco1490 (F) and Hco2198 (R) (Folmer et al., 1994). After iteration up to 100 times with custom sensitivity, a target contig sequence with high coverage was generated. Lastly, the MEGA X (Kumar et al., 2018) was used to check the beginning and end of the contig sequence to circularize a complete mitochondrial genome after deleting the overlapping sequence.

The mitogenome sequence was annotated using MITOS2 webserver (Donath et al., 2019) with invertebrate genetic code. Gene boundaries were confirmed by aligning the gene sequence of the new mitogenome with that of previously reported geometrid mitogenomes available on GenBank with MEGA X (Kumar et al., 2018). The circular maps of the nine mitogenomes generated in this study were comparatively present using the CGView Comparison Tool (Grant et al., 2012). In addition, two species, *Mania lunus* and *Calledapteryx dryopterata*, belonging to Sematuridae and Uraniidae, respectively, were added to the phylogenetic analyses of this study. Mitogenomes of the two species were assembled using the same methods with that of the nine species, from their transcriptomes deposited on GenBank (accession numbers SRR1695439 and SRR1021601).

### 2.3 Sequence alignment and analyses

A total of 38 mitogenomes of Geometroidea were compiled and analyzed, including nine newly sequenced in the present study, two retrieved from transcriptomes publicly published, and 27 downloaded from GenBank. In addition, mitogenomes of 13 species from Noctuoidea, Bombycoidea, Lasiocampoide, Drepanoidea, and Mimallonoidea that represent the close relatives of the Geometroidea were selected as outgroup sequences in phylogenetic analyses (Table 1).

**TABLE 1:** The species used in phylogenetic analyses.

Superfamily	Family	Subfamily	Species	GenBank accession number	Mitogenome size (bp)
Geometroidea	Geometridae	Ennominae	*Abraxas suspecta*	NC_034804	15,537
			*A. latifasciata*	MK962622	15,794
			*Apocheima cinerarium*	NC_024824	15,722
			*A. cinerarius*	KR478686	15,661
			*Biston thoracicaria*	MN956510	15,538
			*B. panterinaria*	NC_020004	15,517
			*B. perclara*.	NC_030769	15,493
			*B. suppressaria*	NC_027111	15,628
			*B. thibetaria*	NC_030632	15,485
			*Ectropis grisescens*	MW337302	15,794
			*E. obliqua*	NC_036717	16,535
			*Hypomecis punctinalis*	MK903031	15,648
			*Milionia basalis*	MN495623	15,901
			*Semiothisa cinerearia*	MK880228	15,523
			***Hydatocapnia marginata***	MZ902340	15,615
			***Luxiaria mitorrhaphes***	MZ902343	15,340
			***Menophra senilis***	MZ902337	15,250
			***Ophthalmitis albosignaria***	MZ902339	15,559
			***Amraica recursaria***	MZ902338	15,582
			***Cotta incongruaria***	MZ902341	15,487
			*Celenna* sp.	KM244697	15,403
			*Erannis ankeraria*	NC_047212	15,250
			*Jankowskia athleta*	NC_027948	15,534
			*Phthonandria atrilineata*	NC_010522	15,499
		Larentiinae	*Dysstroma truncata*	KJ508061	15,828
			*Hydrelia parvulata*	MN962739	15,407
			*Pasiphila chloerata*	MN598218	15,602
			*Operophtera brumata*	NC_027723	15,748
		Geometrinae	*Iotaphora admirabilis*	NC_056092	16,140
			***Pingasa rufofasciata***	MZ902335	15,064
			***Lophophelma iterans***	MZ902342	15,545
		Sterrhinae	*Idaea simplicior*	MN715151	15,950
			*I. effusaria*	MN646772	16,161
			***Somatina indicataria***	MZ902336	15,723
	Epicopeiidae		*Epicopeia hainesii*	MK033610	15,395
	Sematuridae		*Mania lunus*	SRR1695439	
	Uraniidae		*Calledapteryx dryopterata*	SRR1021601	
		Uraniinae	*Lyssa zampa*	MW435592	15,314
Noctuoidea	Erebidae	Erebinae	*Eudocima phalonia*	KY196412	15,575
		Arctiinae	*Hyphantria cunea*	GU592049	15,481
	Noctuidae	Hadeninae	*Mythimna separata*	KM099034	15,329
	Nolidae	Risobinae	*Risoba prominens*	KJ396197	15,343
	Notodontidae	Phalerinae	*Phalera flavescens*	JF440342	15,659
Bombycoidea	Sphingidae	Macroglossinae	*Ampelophaga rubiginosa*	KT153024	15,282
		Sphinginae	*Sphinx morio*	KC470083	15,299
	Saturniidae	Saturniinae	*Eriogyna pyretorum*	FJ685653	15,327
	Bombycidae	Bombycinae	*Bombyx mori*	GU966614	15,656
	Endromidae		*Prismostictoides unihyala*	MF100146	15,355
Lasiocampoidea	Lasiocampidae		*Dendrolimus kikuchii*	MF100138	15,382
Drepanoidea	Drepanidae	Drepaninae	*Drepana arcuata*	KJ508053	15,302
Mimallonoidea	Mimallonidae		*Lacosoma valva*	KJ508050	16,108

*Note*: The species with newly sequenced mitogenome was emphasized in bold.

Among 37 mitochondrial genes, 13 PCGs were individually aligned using the MUSCLE method in the TranslatorX online platform (Abascal et al., 2010) after the sequences were translated with an invertebrate genetic code. Two rRNAs and 22 tRNAs were independently aligned with Q‐INS‐i algorithm as implemented in the MAFFT online platform (Katoh et al., 2019). Further, the aligned tRNA and rRNA sequences were filtered using ClipKIT (Steenwyk et al., 2020) to delete ambiguously aligned sites with the kpic‐gappy algorithm.

Nucleotide composition was calculated using the MEGA X (Kumar et al., 2018). Strand asymmetry was calculated according to the formulas: AT skew = [A − T]/[A + T] and GC skew = [G − C]/[G + C] (Perna & Kocher, 1995). The DAMBE 5.3.74 (Xia, 2013; Xia et al., 2003) was used to conduct tests of substitutional saturation of different data partitions based on the *Iss* (i.e., index of substitutional saturation) statistics. For this method, if *Iss* is positively smaller than *Iss.c* (critical *Iss*), the indicated sequences may have experienced little substitutional saturation (Xia & Lemey, 2009). Nucleotide diversity and the ratio of nonsynonymous substitution (Ka) to synonymous substitution (Ks) for PCGs were calculated using DNASP 5.0 (Librado & Rozas, 2009). The effective number of codon (ENC) was calculated using CodonW 1.4.2 (Peden, 2000).

### 2.4 Phylogenetic analyses

To test the phylogenetic implication of the eleven newly generated mitogenomes, various phylogenetic analyses were performed based on the five following datasets: (1) PCG12: first and second codon positions of 13 PCGs; (2) PCG123: all codon positions of 13 PCGs; (3) PCG12R: first and second codon positions of 13 PCGs plus 24 RNAs; (4) PCG123R: all codon positions of 13 PCGs plus 24 RNAs; (5) PCGAA: amino acid sequences of 13 PCGs.

Maximum likelihood (ML) analyses were conducted using IQ‐TREE 2.0.4 (Nguyen et al., 2015) under the partitioning schemes and corresponding substitution models (Tables S2 and S3) determined by ModelFinder (Kalyaanamoorthy et al., 2017). Branch supports (BS) were calculated using 1000 ultrafast bootstrap replicates (Hoang et al., 2018). Bayesian inference (BI) analyses were performed with MrBayes 3.2.6 (Ronquist et al., 2012) with the partitioned models (Tables S4 and S5) determined by PartitionFinder 2.1.1 (Lanfear et al., 2017). Twelve processors were used to perform two independent runs each with six chains (five heated and one cold) simultaneously for at least 500,000 generations sampled every 100 generations. Convergences were considered to be reached when the estimated sample size (ESS) value was above 200 established by Tracer 1.7 (Rambaut et al., 2018) and the potential scale reduction factor (PSRF) approached 1.0 (Ronquist et al., 2012). The first 25% of samples were discarded as burn‐in and the remaining trees were used to calculate posterior probabilities (PP) in a 50% majority‐rule consensus tree.

## 3 RESULTS AND DISCUSSION

### 3.1 General mitogenome feature and gene rearrangement

Eight complete and one nearly complete mitogenomes were generated and annotated for nine geometrid species, which increased the reported mitogenome diversity, especially for the Geometrinae and Sterrhinae. In the nearly complete genome (*P. rufofasciata*), we failed to assemble the partial sequences of the control region that is characterized by highly biased base composition. The eight completely sequenced mitogenomes ranged from 15,250 bp (*M. senilis*) to 15,723 bp (*S. indicataria*) in size, which are comparable to other reported geometrid mitogenomes (Table 1). All newly generated mitogenomes have been submitted to GenBank with the accession numbers shown in Table 1. The annotation information of mitogenomes sequenced herein is summarized in Table S6. In the two mitogenomes retrieved from transcriptomes, some fragments or genes of RNAs were not assembled, but the 13 PCGs were completely annotated and used only in subsequent phylogenetic analyses.

**FIGURE 1:**
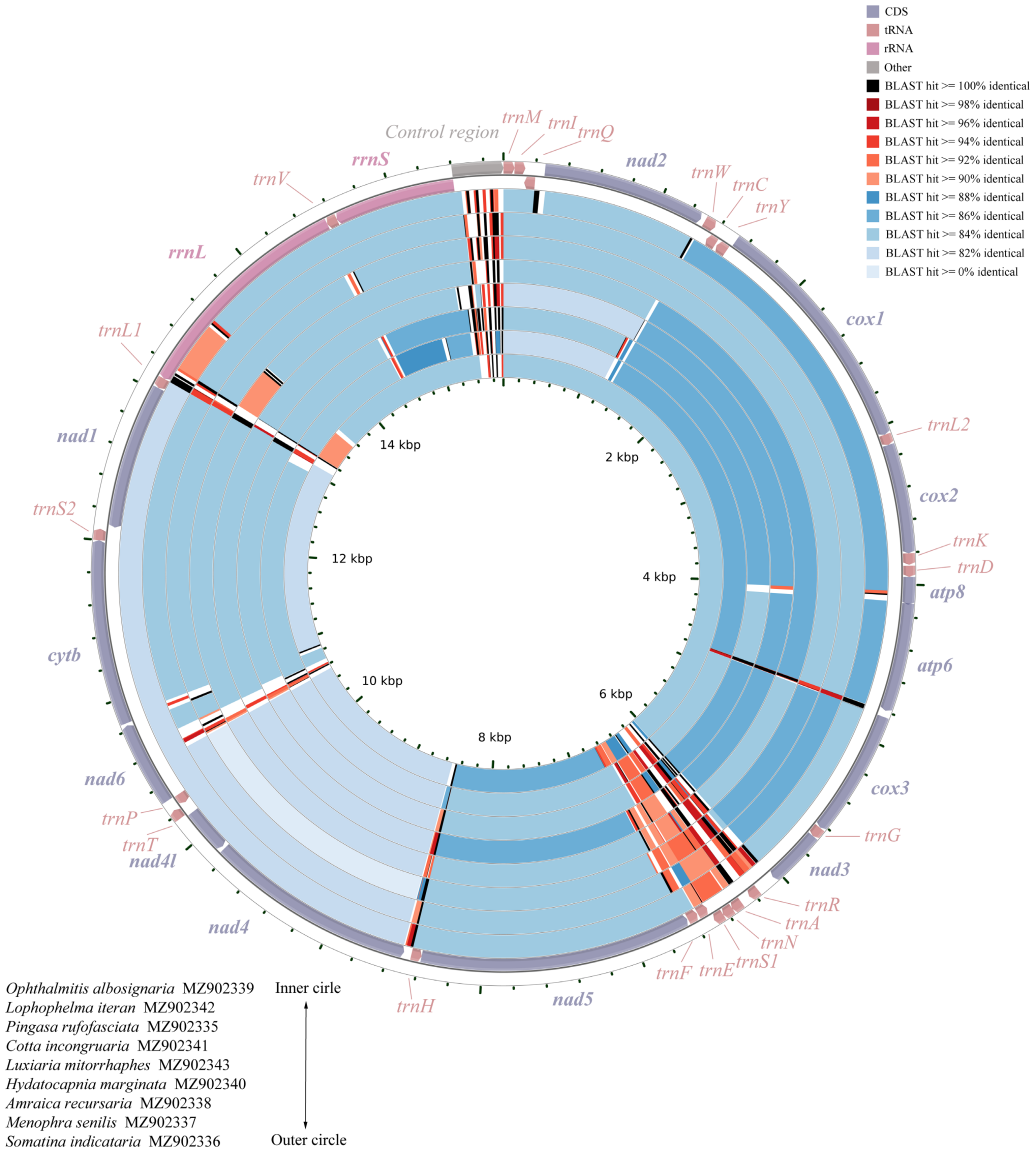
Circular diagram of the nine mitogenomes sequenced in this study. Different color is marked to show the nucleotide identity of BLAST hits relative to the reference mitogenome of *Somatina indicataria* at the outer circle.

**FIGURE 2:**
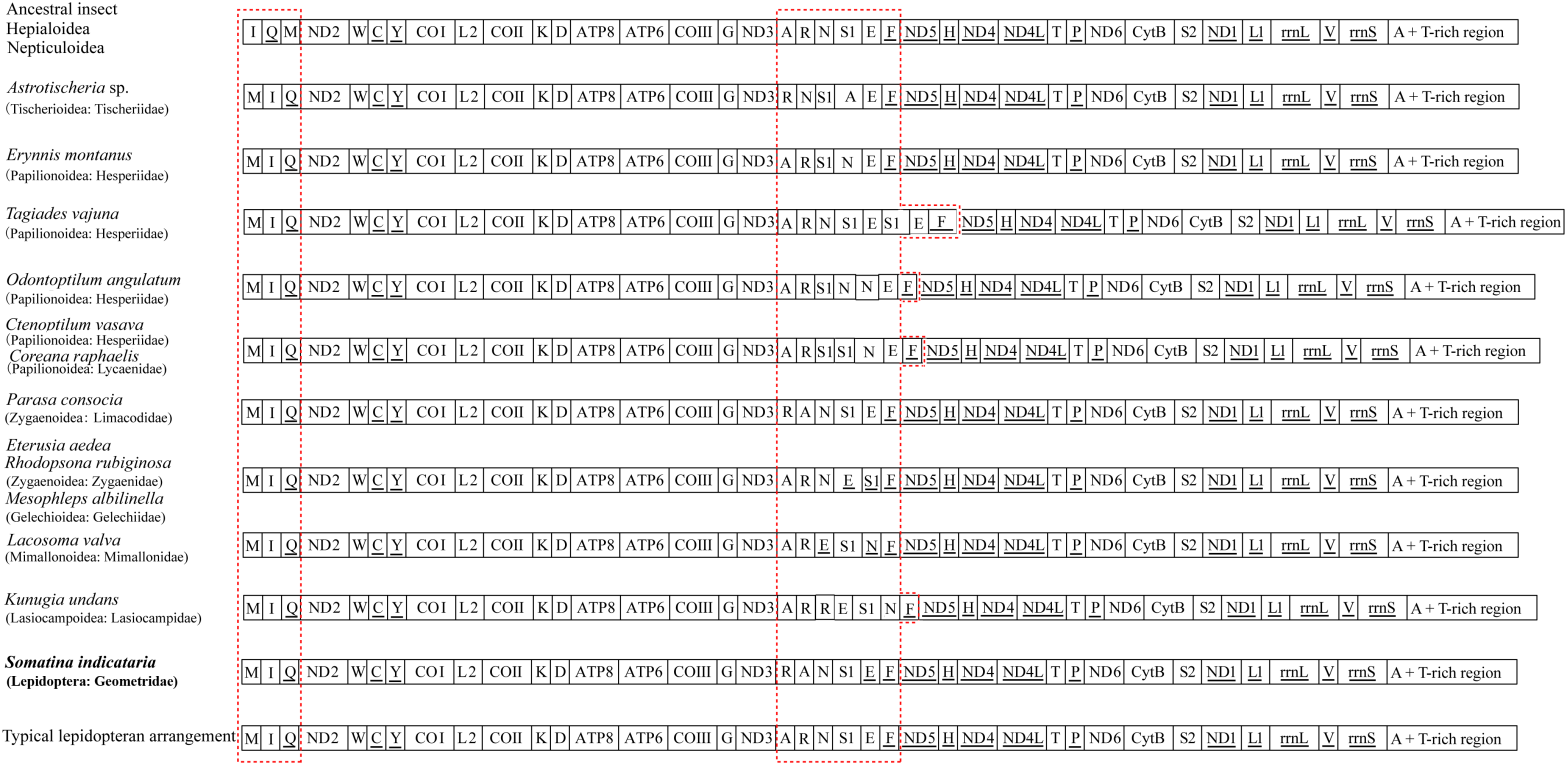
Gene arrangements of reported lepidopteran mitogenomes relative to ancestral insect mitogenome. Red rectangles indicate the two gene clusters with gene rearrangement. The gene with underline is located on the N‐strand. The taxon and its classification with gene rearrangement reported in this study are marked in bold.

All mitogenomes contained 37 mitochondrial genes typical in insects (Figure 1), and these 37 genes, except for *S. indicataria*, showed identical gene organization to other reported geometrid mitogenomes, which are also typical of Lepidoptera (Cameron, 2014; Wu et al., 2016). In the mitogenome of *S. indicataria*, the positions of two tRNAs were arranged. The *trnR* was located before *trnA* instead of after *trnA* typical in Lepidoptera, whereas the *trnE* was translocated from the routinely recognized majority strand (J‐strand) to the minority strand (N‐strand). On the other hand, two long intergenic sequences (121 bp and 61 bp) were present before and after *trnR*, respectively, which were also important features distinct from other reported geometrid mitogenomes. To compare mitogenome evolution, mitochondrial gene rearrangement events previously reported for Lepidoptera were summarized and illustrated in Figure 2. Comparative analysis showed that two rearrangement clusters can be recognized in this order. One includes three tRNAs of *trnM*, *trnI*, and *trnQ*. The gene arrangement *trnM*‐*trnI*‐ *trnQ* is recognized in most lepidopteran members, in contrast to the *trnI*‐ *trnQ* ‐*trnM* in some nonditrysian lineages of Lepidoptera such as the Hepialoidea (Cao et al., 2012). Another is the gene cluster including six tRNAs between *nad3* and *nad5* genes. In this gene cluster, eleven kinds of gene rearrangements have been reported across seven superfamilies. In the *S. indicataria*, the *trnR* is located after *trnA*, similar to the only *Parasa consocia* of Limacodidae in Lepidoptera, whereas the *trnE* was detected rarely on the N‐strand. To confirm this result, we had methodologically reassembled the mitogenome from the high‐throughput sequencing data using Geneious R11 or other software. Moreover, before sequencing, the library was constructed using a single specimen of *S. indicataria*. Overall, the *trnR*‐*trnA*‐*trnN*‐*trnS1*‐ *trnE* ‐ *trnF* recognized in this study represents the first gene rearrangement event reported for Geometroidea and is also unique in Lepidoptera, which broadens our understanding of gene rearrangement in Geometroidea and Lepidoptera.

### 3.2 Nucleotide composition

The A + T content of eight completely sequenced mitogenomes (Table 2) was highly biased, showing 80.9% (*M. senilis*) to 82% (*S. indicataria*), similar to other insect mitogenomes (Boore, 1999). The AT skews were negligible in size (<0.05), but the *S. indicataria* showed a significantly low AT skew (<0.01); by contrast, the GC skews were moderate and comparable below zero between −0.21031 (*A. recursaria*) and −0.17447 (*C. incongruaria*). Overall, the negligible AT skew and moderate GC skew detected in the eight mitogenomes are similar to other Lepidoptera and most insect species (Wei et al., 2010).

**TABLE 2:** Nucleotide composition of nine newly determined mitogenomes for Geometridae.

Subfamily	Species	Mitogenome size (bp)	A%	G%	C%	T%	AT%	AT skew	GC skew
Ennominae	*Menophra senilis*	15,250	41.0	7.7	11.4	39.9	80.9	0.01329	−0.19381
	*Amraica recursaria*	15,582	41.8	7.5	11.4	39.3	81.1	0.03055	−0.21031
	*Ophthalmitis albosignaria*	15,559	41.8	7.5	11.1	39.6	81.4	0.02684	−0.19613
	*Hydatocapnia marginata*	15,615	41.8	7.5	11.4	39.3	81.1	0.03150	−0.20421
	*Cotta incongruaria*	15,487	41.5	7.7	10.9	39.9	81.4	0.01999	−0.17447
	*Luxiaria mitorrhaphes*	15,340	41.0	7.7	11.2	40.0	81.1	0.01262	−0.18567
Geometrinae	*Pingasa rufofasciata*	15,064	41.2	8.0	11.6	39.2	80.4	0.02469	−0.18253
	*Lophophelma iterans*	15,545	42.5	7.7	11.0	38.8	81.3	0.04534	−0.17481
Sterrhinae	*Somatina indicataria*	15,723	41.2	7.4	10.6	40.9	82.0	0.00395	−0.17976

**FIGURE 3:**
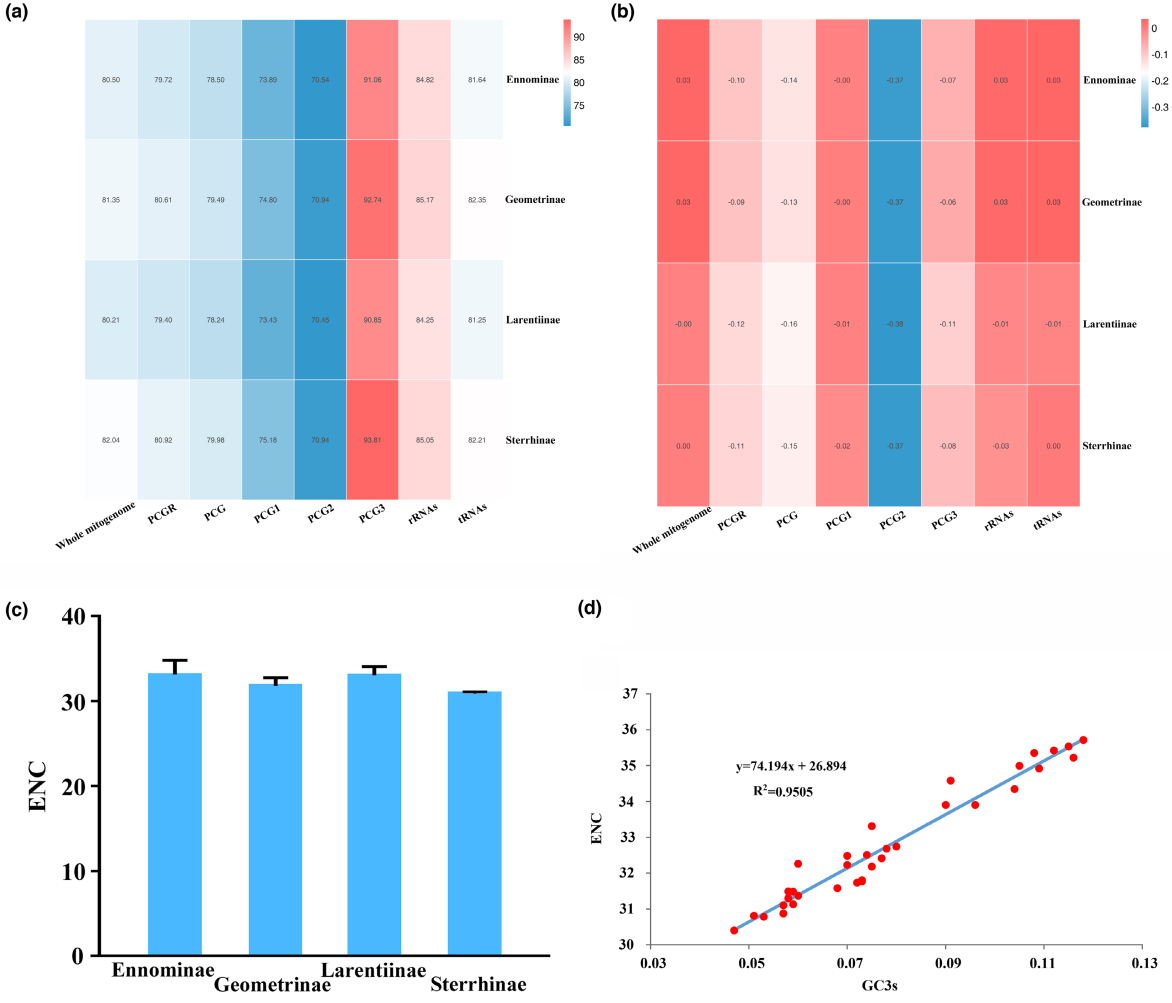
Nucleotide composition of the geometrid mitogenomes. (a) A + T content. (b) AT skew. (c) The averaged effective number of codons (ENC) of four geometrid subfamilies. (d) Scatter plot of the GC content of 3rd codon sites versus ENC.

Among the four subfamilies of Geometridae (Figure 3a), the A + T contents ranged from 80.21% (Larentiinae) to 82.04% (Sterrhinae), showing little heterogeneity in nucleotide composition, which is in contrast to some insect groups generally at the same taxonomic levels (Liu et al., 2018; Nie et al., 2020; Song et al., 2016; Tang et al., 2019; Yang et al., 2018). Among the three codon positions within the 13 PCGs, the lowest A + T content was found for the second codon position, followed by the first and third codon positions, in accordance with most groups of insects, such as Zygaenoidea of Lepidoptera (Yang, Song, et al., 2019; Yang, Zhang, et al., 2019) and Cimicomorpha of Hemiptera (Yang et al., 2018). Overall, rRNAs showed a higher A + T content than PCGs and tRNAs. The AT skew and GC skew are commonly used for evaluating the nucleotide composition of insect mitogenomes (Perna & Kocher, 1995; Wei et al., 2010). In Geometridae, negligible AT skews and negative GC skews were recognized (Figure 3b, Table S7), and four geometrid subfamilies consistently showed that the second codon positions of 13 PCGs and rRNAs had the lowest values of AT skew and GC skew, respectively, a feature commonly present in other lepidopteran families (Cameron & Whiting, 2008) such as the Tortricidae recently reported by Yang et al. (2021). The ENC is routinely regarded between 20 and 61 and is negatively correlated with codon usage bias. The ENC = 20 indicates an absolute bias toward a synonymous codon, whereas ENC = 61 indicates the neutral codon usage (Wright, 1990). In the reported mitogenomes of Geometridae, the ENC values (Figure 3c) ranged from 30.4 to 35.53 and have almost no difference among the four subfamilies involved in this study but overall exhibiting codon usage bias to some extent. Moreover, the positive correlation between the ENC and GC3s (Figure 3d) indicates that the genomic G + C content is a significant factor in determining codon bias among geometrid species (Hershberg & Petrov, 2008; Plotkin & Kudla, 2011).

### 3.3 Mitochondrial gene variation of Geometridae

**FIGURE 4:**
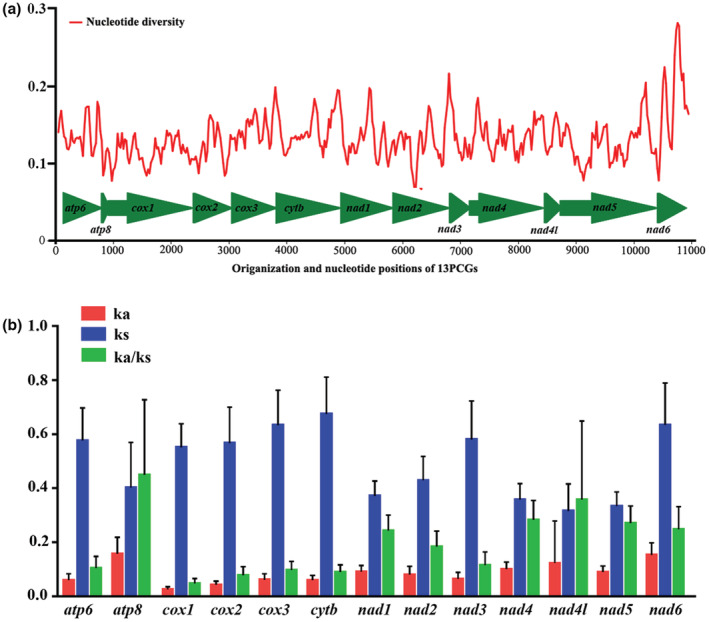
Gene variation of 13 PCGs in Geometridae. (a) The sliding window analysis shows the value of nucleotide diversity. (b) The Ka, Ks, and Ka/Ks of each PCG among geometrid representatives. Ka—nonsynonymous substitution; Ks—synonymous substitution.

Nucleotide diversity is commonly used for identifying regions with high nucleotide divergence and could provide guidelines for selecting species‐ or group‐specific markers used in molecular evolutionary studies, especially for taxa with high morphological similarity (Jia et al., 2010; Ma et al., 2020). To evaluate the variation patterns of 13 PCGs of Geometridae, nucleotide diversity was calculated through sliding window analysis for each PCG. The results (Figure 4a) showed a variable nucleotide diversity both within and among PCGs. At the gene level, the average values of nucleotide diversity varied from 0.114 (*cox1*) to 0.199 (*nad6*). The gene with nucleotide diversity next to nad6 was *atp8*, followed by *cytb*, *cox3*, *nad4l*, and *nad4*. The average values of nucleotide diversity for 13 PCGs can be also indicated by the sliding window analysis. The genes or gene regions with higher levels of nucleotide diversity identified herein could provide potential marker candidates for population genetics and species delimitation in Geometridae. In addition, the values of Ka, Ks, and Ka/Ks were calculated to compare the evolutionary patterns of 13 PCGs. As shown in Figure 4b, the *atp8* and *cox1* exhibit the highest and lowest Ka/Ks rates, respectively, indicating the 13 PCGs have different evolutionary rates. Notably, the Ka/Ks values for all PCGs were lower than one, indicating that they are evolving under purifying selection and are suitable for investigating phylogenetic relationships within the Geometridae.

### 3.4 Phylogenetic analyses of Geometroidea

Tests of substitution saturation (Table 3) showed all *Iss* values in the first and second coding positions of 13 PCGs and 22 tRNA were significantly lower than *Iss.c* values for both symmetrical and asymmetrical topologies. By contrast, the third coding positions of 13 PCGs and two RNAs showed evolutionary saturation to some extent, indicating that they had a faster evolutionary rate and might contain phylogenetic noise information (Owen et al., 2015). Thus, in subsequent phylogenetic analyses, five datasets associated with the inclusion and exclusion of the third coding positions and RNA sequences were considered to test the stability of topologies.

**TABLE 3:** Saturation tests of different data partitions.

	NumOTU	*Iss*	*Iss.cSym*	*PSym*	*Iss.cAsym*	*PAsym*
PCG1s	32	0.286	0.809	0.0000	0.554	0.0000
PCG2s	32	0.165	0.809	0.0000	0.554	0.0000
PCG3s	32	0.595	0.809	0.0000	0.554	0.0000
rRNAs	32	0.768	0.797	0.4301	0.531	0.0000
tRNAs	32	0.424	0.777	0.0000	0.496	0.0455

*Note*: Two‐tailed tests were used.

**FIGURE 5:**
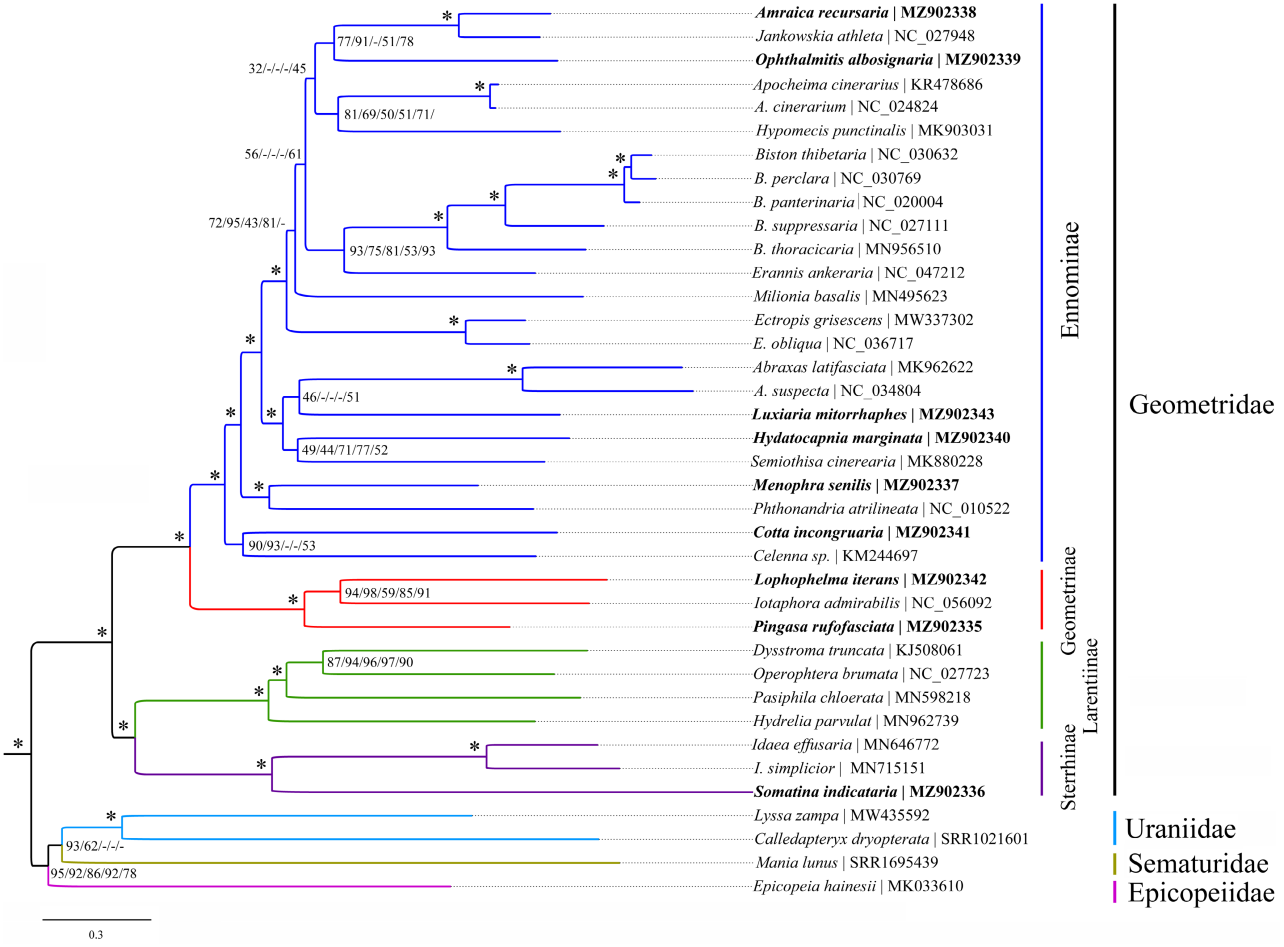
Maximum likelihood (ML) tree inferred from IQ‐TREE method based on PCG123R dataset. The species with newly sequenced mitogenome is emphasized in bold. Numbers separated by slash (/) on node represent the bootstrap values based on the PCG123R, PCG123, PCG12R, PCG12, and PCGAA datasets, respectively. The “*” on node represents bootstrap values ≥90 for all datasets. The “‐” represents an unrecovered node in ML tree of the corresponding dataset.

**FIGURE 6:**
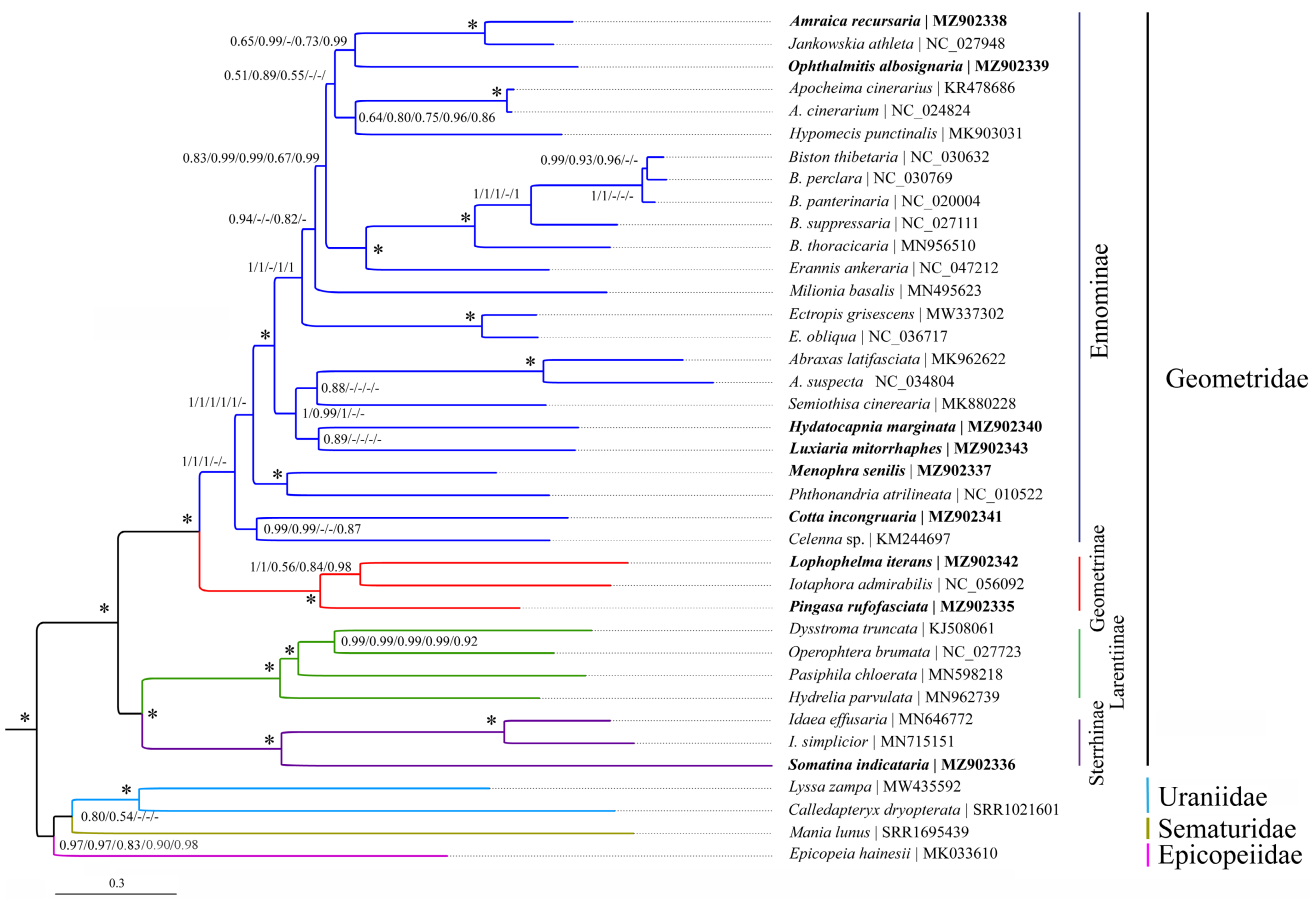
Bayesian tree inferred from MrBayes method based on PCG123R dataset. The species with newly sequenced mitogenome is emphasized in bold. Numbers separated by slash (/) on node represent the posterior probabilities based on the PCG123R, PCG123, PCG12R, PCG12, and PCGAA datasets, respectively. The “*” on node represents posterior probabilities ≥0.95 for all datasets. The “‐” represents unrecovered node in Bayesian tree of corresponding dataset.

According to recent molecular investigations (Bazinet et al., 2013; Rajaei et al., 2015; Regier et al., 2013; Wang et al., 2019; Yang, Zhang, et al., 2019), five families are included in Geometroidea. The present study sampled four families, including the Uraniidae represented in the mitogenome‐based phylogenetic investigation for the first time. Based on the hitherto most extensive mitogenomic sampling, our various resulting trees (Figures 5 and 6) showed generally the same topologies especially in terms of family‐ and subfamily‐level relationships. The relationships among four families were recovered as Geometridae + (Epicopeiidae + (Uraniidae + Sematuridae)) or Geometridae + (Sematuridae + (Epicopeiidae + Uraniidae)), with either Epicopeiidae or Sematuridae being sister to Uraniidae depending on different datasets. In detail, in ML analyses, the datasets PCG123R and PCG123 yielded the Uraniidae + Sematuridae, whereas other datasets showed Epicopeiidae + Uraniidae. In Bayesian analyses, the same situation was recovered. In terms of dataset selection, the results above indicate that the third coding positions of 13 PCGs have a deep effect on the tree topologies under both inference methods, whereas RNA genes did not. In addition, the tree based on PCGAA dataset showed a similar topology with that of PCG12R and PCG12 datasets. These results overall indicate that the third coding positions of 13 PCGs contain high phylogenetic informativeness although they may have experienced some substitution saturation (Yang et al., 2021). Regardless of the Pseudobistonidae with no mitogenome available, the close relationship between Epicopeiidae and Sematuridae is recovered by multilocus data in previous molecular studies (Rajaei et al., 2015; Wang et al., 2019). However, the placement of Uraniidae remains controversial, being closer to the Epicopeiidae and Sematuridae rather than to Geometridae as recovered by most of the previous multilocus molecular studies but with low to moderate supports (e.g., Rajaei et al., 2015; Wang et al., 2019). In this study, based on mitogenome evidence for the first time, the relationships among Sematuridae, Epicopeiidae, and Uraniidae remained unresolved, indicating that further investigation is necessary based on extensive sampling of these families (Mitter et al., 2017).

In Geometridae, eight subfamilies are currently recognized (Murillo‐Ramos et al., 2019; Sihvonen et al., 2011). The present study sampled representatives of four subfamilies and their relationships were consistently recovered as (Sterrhinae + Larentiinae) + (Geometrinae + Ennominae) with strong supports. This topology is accordant with that of a multilocus study (Murillo‐Ramos et al., 2019) regardless of other four subfamilies with no mitogenome available.

The nine mitogenomes sequenced in the present study represented nine species of three subfamilies of the Geometridae, of which one belongs to Sterrhinae, two from Geometrinae, and the remaining six from Ennominae. Their taxonomic assignments were confirmed using mitogenome evidence for the first time, which provide support for previous morphological studies (Han & Xue, 2011; Jiang et al., 2011, 2012, 2014; Kuzmin & Beljaev, 2021; Sihvonen & Kaila, 2004; Walia, 2015).

## AUTHOR CONTRIBUTIONS

**Weili Ding:** Conceptualization (equal); software (equal); validation (equal); writing – original draft (lead). **Haizhen Xu:** Methodology (equal); software (equal). **Zhipeng Wu:** Methodology (equal); software (equal). **Lizong Hu:** Conceptualization (equal); methodology (equal); writing – review and editing (equal). **Li Huang:** Conceptualization (equal); methodology (equal); writing – review and editing (equal). **Mingsheng Yang:** Conceptualization (equal); funding acquisition (equal); validation (equal); writing – review and editing (supporting). **Lili Li:** Conceptualization (equal); funding acquisition (equal); project administration (lead); supervision (lead); validation (equal); writing – review and editing (equal).

## CONFLICT OF INTEREST STATEMENT

The authors declare no conflict of interest.

## Supporting information


Table S1.

Table S2.

Table S3.

Table S4.

Table S5.

Table S6.

Table S7.

## Data Availability

All mitogenome sequences generated in this study were deposited in the GenBank under accession numbers MZ902335–MZ902343.
