# Supplementary material for: The mitochondrial genomes of the Geometroidea (Lepidoptera) and their phylogenetic implications
Source: Ecol Evol. 2023 Feb 9;13(2):e9813. doi: 10.1002/ece3.9813 (PMC9911631; doi:10.1002/ece3.9813)
Supplement: Supplementary file 1 — Table S1. [file ECE3-13-e9813-s006.docx]

**Table S1 Information of samples with mitogenomes sequenced in this study**

| **Family** | **Subfamily** | **Species** | **Specimen acc.** | **Locality** | **Longitude/latitude** | **Collection time** |
| --- | --- | --- | --- | --- | --- | --- |
| Geometridae | Ennominae | *Hydatocapnia marginata* | Jgs2020138M | Xinyang, Henan, China | 114°03′59″E/31°49′14″N | Jul. 2020 |
|  |  | *Luxiaria mitorrhaphes* | Pg202098M | Lushan, Henan, China | 112°30′56″E/33°41′00″N | Aug. 2020 |
|  |  | *Menophra senilis* | Jgs2020273M | Xinyang, Henan, China | 114°03′59″E/31°49′14″N | Jul. 2020 |
|  |  | *Ophthalmitis albosignaria* | Jgs202051M | Xinyang, Henan, China | 114°03′59″E/31°49′14″N | Jul. 2020 |
|  |  | *Amraica recursaria* | Jgs2020277M | Xinyang, Henan, China | 114°03′59″E/31°49′14″N | Jul. 2020 |
|  |  | *Cotta incongruaria* | Jgs2020207M | Xinyang, Henan, China | 114°03′59″E/31°49′14″N | Jul. 2020 |
|  | Geometrinae | *Lophophelma iterans* | Lzy202090M | Lushan, Henan, China | 112°31′20″E/33°44′19″N | Aug. 2020 |
|  |  | *Pingasa rufofasciata* | Jgs2020149M | Xinyang, Henan, China | 114°03′59″E/31°49′14″N | Jul. 2020 |
|  | Sterrhinae | *Somatina indicataria* | Jgs20248M | Xinyang, Henan, China | 114°03′59″E/31°49′14″N | Jul. 2020 |
