# Supplementary material for: The mitochondrial genomes of the Geometroidea (Lepidoptera) and their phylogenetic implications
Source: Ecol Evol. 2023 Feb 9;13(2):e9813. doi: 10.1002/ece3.9813 (PMC9911631; doi:10.1002/ece3.9813)
Supplement: Supplementary file 2 — Table S2. [file ECE3-13-e9813-s003.docx]

Table S2. The partitioning schemes and corresponding substitution models determined by ModelFinder for the PCG123R dataset

| Partitions | Model | Data partitions |
| --- | --- | --- |
| P1 | GTR+F+I+G4 | a6p1,c3p1, cbp1 |
| P2 | TVM+F+R3 | a6p2, c1p2, c2p2,c3p2, cbp2 |
| P3 | TPM2+F+R5 | a6p3, c1p3, c2p3 |
| P4 | GTR+F+I+G4 | a8p1, n2p1, n3p1, n6p1 |
| P5 | TVM+F+R3 | a8p2, n2p2, n3p2, n6p2 |
| P6 | GTR+F+R4 | a8p3, n2p3 |
| P7 | GTR+F+I+G4 | c1p1, c2p1 |
| P8 | TIM+F+R5 | c3p3, cbp3, n3p3, n6p3 |
| P9 | TVM+F+R4 | n1p1, n4p1, n4lp1, n5p1 |
| P10 | GTR+F+R4 | n1p2, n4p2, n4lp2, n5p2 |
| P11 | TIM+F+R6 | n1p3, n4p3, n4lp3, n5p3 |
| P12 | GTR+F+R4 | *rrnS, rrnL* |
| P13 | GTR+F+R4 | tRNAs |

Note: c1–c3, n1–n6, a6, a8 and cb indicate the 13 PCGs; the p1, p2 and p3 indicate the first,second and third codon positions of each PCG respectively.
