# Supplementary material for: The mitochondrial genomes of the Geometroidea (Lepidoptera) and their phylogenetic implications
Source: Ecol Evol. 2023 Feb 9;13(2):e9813. doi: 10.1002/ece3.9813 (PMC9911631; doi:10.1002/ece3.9813)
Supplement: Supplementary file 3 — Table S3. [file ECE3-13-e9813-s005.docx]

Table S3. The partitioning schemes and corresponding substitution models

determined by ModelFinder for the PCGAA dataset

| Partitions | Models | Amino acid partitions |
| --- | --- | --- |
| P1 | mtMet+R4 | ATP6, COX1, COX2, COX3, CYTBAA |
| P2 | mtMet+F+R5 | ATP8, ND2, ND3, ND6 |
| P3 | mtZOA+F+R5 | ND1, ND4, ND4L, ND5 |
