# Supplementary material for: The mitochondrial genomes of the Geometroidea (Lepidoptera) and their phylogenetic implications
Source: Ecol Evol. 2023 Feb 9;13(2):e9813. doi: 10.1002/ece3.9813 (PMC9911631; doi:10.1002/ece3.9813)
Supplement: Supplementary file 4 — Table S4. [file ECE3-13-e9813-s004.docx]

Table S4. The partitioning schemes and corresponding substitution models

determined by PartitionFinder for the PCG123R dataset

| Partitions | Model | Data partitions |
| --- | --- | --- |
| P1 | GTR+I+G | a6p1, c3p1, cbp1 |
| P2 | TVM+I+G | a6p2, c1p2, c2p2, c3p2, cbp2 |
| P3 | TRN+I+G | a6p3, c3p3, n3p3, n6p3, cbp3 |
| P4 | GTR+I+G | a8p1, a8p2, n2p1, n3p1, n6p1 |
| P5 | K81UF+G | a8p3, n2p3 |
| P6 | GTR+I+G | c1p1, c2p1 |
| P7 | HKY+G | c1p3, c2p3 |
| P8 | TVM+I+G | n1p1, n4p1, n4lp1, n5p1 |
| P9 | GTR+I+G | n1p2, n4p2, n4lp2, n5p2 |
| P10 | TIM+G | n1p3, n4lp3 |
| P11 | TVM+I+G | n2p3, n3p3, n6p2 |
| P12 | GTR+I+G | n4p3, n5p3 |
| P13 | GTR+I+G | *rrnS, rrnL* |
| P14 | GTR+I+G4 | tRNAs |
