# Supplementary material for: The mitochondrial genomes of the Geometroidea (Lepidoptera) and their phylogenetic implications
Source: Ecol Evol. 2023 Feb 9;13(2):e9813. doi: 10.1002/ece3.9813 (PMC9911631; doi:10.1002/ece3.9813)
Supplement: Supplementary file 5 — Table S5. [file ECE3-13-e9813-s007.docx]

Table S5. The partitioning schemes and corresponding substitution models

determined by PartitionFinder for the PCGAA dataset

| Partitions | Models | Amino acid partitions |
| --- | --- | --- |
| P1 | MTART+I+G+F | ND2, ND3, CYTB, ATP6 |
| P2 | MTREV+G+F | ND6, ATP8 |
| P3 | MTART+I+G | COX1 |
| P4 | MTART+I+G | COX2, COX3 |
| P5 | MTART+I+G+F | ND4L, ND4, ND5, ND1 |
