# Supplementary material for: The mitochondrial genomes of the Geometroidea (Lepidoptera) and their phylogenetic implications
Source: Ecol Evol. 2023 Feb 9;13(2):e9813. doi: 10.1002/ece3.9813 (PMC9911631; doi:10.1002/ece3.9813)
Supplement: Supplementary file 7 — Table S7. [file ECE3-13-e9813-s001.docx]

**Table S6. GC skew of the geometrid mitogenomes**

|  | Ennominae | Geometrinae | Larentiinae | Sterrhinae |
| --- | --- | --- | --- | --- |
| Whole mitogenome | -0.207 | -0.174 | -0.195 | -0.189 |
| PCGR | -0.019 | -0.006 | -0.029 | -0.014 |
| PCG | 0.038 | 0.052 | 0.027 | 0.047 |
| PCG1 | 0.261 | 0.266 | 0.240 | 0.253 |
| PCG2 | -0.096 | -0.093 | -0.102 | -0.094 |
| PCG3 | -0.170 | -0.111 | -0.177 | -0.120 |
| rRNAs | -0.351 | -0.333 | -0.357 | -0.345 |
| tRNAs | -0.133 | -0.128 | -0.135 | -0.134 |
